# Interaction of lifeguard (LFG/FAIM2) with phosphoinositides is associated with altered intracellular calcium homeostasis in breast cancer cells

**DOI:** 10.1016/j.bbrep.2026.102830

**Published:** 2026-10-09

**Authors:** Anna Marie Welzel, Lena Maria Herrmann, Nadjib Dastagir, Sarah Strauß, Peter M. Vogt, Vesna Bucan

**Affiliations:** Department of Plastic, Aesthetic, Hand and Reconstructive Surgery, Hanover Medical School, Carl-Neuberg-Strasse 1, Hannover, D 30625, Germany

**Keywords:** LFG (FAIM2), Breast cancer, Phosphoinositides, Intracellular calcium homeostasis, Apoptosis

## Abstract

Lifeguard (LFG/FAIM2) is an anti-apoptotic transmembrane protein implicated in apoptosis resistance and breast cancer progression. However, the molecular mechanisms underlying its biological function remain incompletely understood.

In the present study, we investigated potential interactions between LFG and phosphoinositides in the breast cancer cell line MDA-MB-231. Using membrane lipid strip and phosphoinositide (PIP) array assays, we observed binding signals between LFG and several phosphoinositide species, indicating potential lipid-binding preferences under the experimental conditions employed.

Because phosphoinositides participate in intracellular calcium signaling pathways, we next examined whether reduced LFG expression was associated with alterations in intracellular calcium homeostasis. Using a fluorescence-based calcium assay, we found that siRNA-mediated downregulation of LFG was associated with increased intracellular calcium-associated fluorescence.

Overall, our exploratory findings suggest that LFG may participate in phosphoinositide-associated signaling and intracellular calcium homeostasis in the breast cancer cell model examined. Further studies will be required to validate these observations, determine the underlying molecular mechanisms, and assess their potential relevance for apoptosis regulation and breast cancer progression.

## Highlights

• The function of Lifeguard (LFG), an anti-apoptotic transmembrane protein, in breast carcinoma cells still remains to be fully understood.
• Differential LFG-Associated binding signals to different phospoinositides provide preliminary evidence of potential lipid-binding preferences.
• The downregulation of LFG leads to increased intracellular Ca^2+^ levels in MDA-MB-231 breast cancer cells.
• Results suggest a potential link between LFG, phosphoinositide-associated signaling and intracellular calcium homeostasis in breast cancer cells, providing a basis for future investigation.

## 1 Introduction

Although therapeutic options for breast cancer continue to evolve, it remains one of the most common and deadly malignancies in women [1,2]. With a better understanding of the underlying mechanisms of the molecular pathways involved in carcinogenesis, new opportunities for both diagnosis and treatment can be identified [3,4]. A key process controlling cancer cell progression is the regulation of programmed cell death, or apoptosis. Apoptotic pathways, which normally play a critical role in regulating cellular damage and act as a natural barrier to cancer, can contribute to tumor development and growth when the physiological process is disrupted, resulting in tumor cells that acquire resistance to cell death [[5], [6], [7], [8], [9], [10]].

The mammary gland is constantly undergoing dynamic changes regulated by the balance between pro-apoptotic and anti-apoptotic proteins. This balance is critical for maintaining homeostasis between normal and damaged cells [11,12]. Numerous anti-apoptotic proteins have been identified as influencing carcinogenesis, including the lifeguard protein (LFG, also known as Fas apoptotic inhibitory molecule 2/FAIM2), which has been found to be highly expressed in breast cancer cells and tissues [13]. This highly conserved member of the transmembrane Bax inhibitor 1 motif-containing (TMBIM) protein family has been studied for its multi-transmembrane structure, widespread expression in healthy and pathological tissues, and membrane-associated localization in the endoplasmic reticulum (ER), plasma membrane, Golgi and vesicular compartments [[13], [14], [15], [16], [17], [18], [19], [20], [21], [22], [23]]. Furthermore, interactions with the Fas/FasL apoptosis pathway, regulation of apoptotic signaling, calcium (Ca^2+^) homeostasis, ER stress signaling and autophagy have been described in previous studies [[13], [14], [15], [16], [17], [18], [19], [20], [21], [22], [23]].

While the anti-apoptotic effect of LFG is well documented, the specific signaling pathways and molecular mechanisms contributing to its anti-apoptotic function remain poorly characterized [[15], [16], [17]]. Previous studies have associated LFG and other members of the TMBIM family with alterations in intracellular calcium homeostasis and ER-associated calcium signaling. However, the precise mechanisms by which LFG itself contributes to these processes remain incompletely understood [24,25].

Calcium release from the ER into the cytoplasm plays a critical role in apoptosis, leading to caspase activation, cytochrome *c* release and further apoptotic signaling [[26], [27], [28], [29]]. An important calcium-release channel involved in these processes is the inositol 1,4,5-triphosphate receptor (IP3R), which releases calcium into the cytoplasm in response to its activating second messenger, inositol 1,4,5-triphosphate (IP3) [[30], [31], [32]]. This release of calcium is essential not only for the intrinsic apoptotic pathway but also for the extrinsic Fas-induced apoptotic pathway, thereby increasing cellular sensitivity to apoptosis [29,33,34]. Previous studies have shown that high expression of certain anti-apoptotic proteins of the Bcl-2 family correlates with reduced levels of IP3R and calcium release [35,36]. However, no direct interaction or binding between IP3R and LFG has been demonstrated, and the molecular relationship between LFG and IP3R-dependent calcium signaling therefore remains unclear [37].

Members of the TMBIM protein family, including LFG, have been implicated in the regulation of intracellular calcium homeostasis and ER calcium dynamics [24,38]. However, the molecular mechanisms appear to differ between individual TMBIM family members, and findings obtained for other members of this protein family cannot necessarily be directly transferred to LFG. This suggests that LFG may contribute to the regulation of Ca^2+^ homeostasis, although the underlying mechanism remains to be established.

Several members of the TMBIM family have been reported to influence ER calcium permeability or calcium leakage [24,38]. Whether LFG itself possesses comparable calcium-permeability properties, however, has not been conclusively established. Therefore, potential effects of LFG on intracellular calcium homeostasis should be distinguished from the better-characterized calcium-regulatory mechanisms described for other TMBIM family members [4,38].

Given the critical role of phosphoinositide signaling in intracellular calcium regulation, potential interactions between LFG and components of the phosphoinositide signaling system remain of interest. Phosphoinositides are located at the plasma membrane as well as in distinct intracellular membrane compartments, where they are dynamically phosphorylated and dephosphorylated according to the functional requirements of the cell [[39], [40], [41], [42], [43], [44]]. Phosphoinositides play many roles, including cell signaling, membrane organization, cytoskeletal remodeling and inter-organelle communication through membrane contact sites and lipid-protein binding, all of which influence cellular homeostasis [[39], [40], [41], [42], [43], [44], [45], [46]].

Within the canonical phospholipase C (PLC)-dependent calcium signaling pathway, phosphatidylinositol 4,5-bisphosphate (PtdIns(4,5)P_2_) serves as the principal substrate for PLC and is hydrolyzed to generate IP3 and diacylglycerol (DAG). IP3 subsequently binds to IP3R on the ER and promotes intracellular calcium release [32,39,47,48]. By contrast, phosphatidylinositol (3,4,5)-trisphosphate (PtdIns(3,4,5)P_3_) is primarily generated through PI3K-dependent phosphorylation of PtdIns(4,5)P_2_ and functions predominantly in PI3K/AKT-associated signaling pathways [45,46,[49], [50], [51]]. Although these phosphoinositide pathways are interconnected, an interaction with PtdIns(3,4,5)P_3_ does not by itself imply altered PLC activity, IP3 production, or IP3R-mediated calcium release.

Previous studies from our group demonstrated that LFG is significantly overexpressed in breast cancer tissues and breast cancer cell lines compared with normal mammary tissue and contributes to apoptosis resistance in malignant cells [13,14]. Although increased LFG expression has been reported in breast cancer, current evidence does not indicate that this expression is restricted exclusively to triple-negative breast cancer (TNBC). MDA-MB-231 cells were selected for the present study because they represent a well-established TNBC model exhibiting relatively high endogenous LFG expression and are therefore particularly suitable for siRNA-mediated knockdown studies investigating LFG function [13,16]. In this study, we investigated potential interactions between LFG and membrane lipids, particularly phosphoinositides, and examined whether reduced LFG expression was associated with alterations in intracellular calcium homeostasis in MDA-MB-231 breast cancer cells.

## 2 Methods and material

### 2.1 Cell line and culture condition

The human breast cancer cell line MDA-MB-231, a commonly used model for triple negative breast cancer research, was obtained from the American Type Culture Collection (ATCC), Rockville, MD, USA. These cells were cultured in Dulbecco's modified Eagle's medium (DMEM) obtained from PAA Laboratories GmbH, Cölbe, Germany. The medium was supplemented with 10% fetal calf serum (FCS) purchased from Biochrom, Berlin, Germany. MDA-MB-231 cells were maintained at a constant temperature of 37°C in a humidified incubator with a controlled atmosphere of 5% CO_2_.

For routine maintenance and expansion, cells were subcultured every 2 to 3 days. In this process, the cells were detached from the culture flask using a 0.25% trypsin/0.53 mM ethylenediaminetetraacetic acid (EDTA) solution.

### 2.2 Small interfering RNA

The MDA-MB-231 human breast cancer cell line was subjected to specific genetic manipulation to investigate the role of a target gene. The cells were transfected with ON-TARGETplus Human FAIM2 (LFG) siRNA purchased from Sirion Biotech, Munich, Germany. An appropriate control sequence was also used to ensure that any observed effects were specifically due to the siRNA-mediated knockdown and not to other transfection-related factors.

The design of the siRNA was optimized using an algorithm developed by Sirion Biotech. This algorithm focuses on maximizing gene specificity and knockdown (KD) efficiency. Specificity ensures that the siRNA selectively targets the LFG gene without off-target effects, while efficiency refers to the ability of the siRNA to effectively reduce the expression of the target gene.

For the transfection process, the MDA-MB-231 cells were seeded at a density of 2 × 10^4^ cells per square centimeter. After seeding, the cells were incubated under standard cell culture conditions (i.e. at 37°C in a humidified atmosphere with 5% CO_2_). After 5 min of incubation at room temperature, a mix of 6 μM of the siRNA sequence, 200 μl of medium and 6 μl of Lipofectamine was added to each 25 cm^3^ flask of breast cancer cells. The incubation times were set at 24 and 48 h to allow sufficient time for the siRNA to take effect. Knockdown efficiency was verified by Western blot and quantified by densitometry using ImageJ.

### 2.3 Western blot analysis

To investigate the effects of siRNA-mediated gene knockdown on protein expression, total protein extracts were prepared from MDA-MB-231 cell cultures. These cultures were incubated with LFG-650 siRNA and corresponding control treatments for 24 and 48 h. After incubation, the cells were lysed to extract total proteins using Radio-Immunoprecipitation Assay (RIPA) buffer. RIPA Buffer is a potent lysis buffer that efficiently solubilizes cellular proteins while inhibiting protease activity to prevent protein degradation.

Subsequently, 25 μg of total protein from each sample was separated by electrophoresis on 15% SDS-polyacrylamide gels. This high percentage gel is suitable for resolving lower molecular weight proteins. After electrophoresis, the proteins were transferred to a polyvinylidene fluoride (PVDF) membrane (Millipore Corporation) to facilitate subsequent immunoblotting. Immunoblotting was performed using specific polyclonal antibodies to detect the proteins of interest. The antibodies used were:

rabbit polyclonal anti-hLFG antibody (1:500 dilution; Novusbiologicals, Wiesbaden Nordenstadt, Germany) and anti-beta-Tubulin (1:500 dilution; Stemcell Technologies, Vancouver, British Columbia, Canada). After incubation with the primary antibodies, the membranes were treated with Odyssey 680/800 nm secondary conjugates (Li-Cor BioSciences, Lincoln, NE, USA). These secondary antibodies are conjugated to fluorescent dyes, enabling the quantification of protein expression levels by fluorescence detection. Protein signals were visualized using the Odyssey Infrared Imaging System and the accompanying software from LI-COR Biosciences. The band intensities were subsequently determined by densitometric analysis using ImageJ, normalized to β-tubulin.

### 2.4 Ca^2+^-assay

Metabolic activity of viable cells based on intracellular calcium mobilization was assessed using the Screen Quest™ Calbryte-520 Assay Kit (Promega, Madison, WI, USA) according to the manufacturer's instructions. MDA-MB-231 cells were seeded at a density of 10^4^ cells per well in two separate 96-well plates. Cells were incubated for 24 h at 37°C in a humidified atmosphere containing 5% CO_2_ to allow for cell adhesion and growth. Following the initial 24-h incubation period, the cells were transfected with LFG siRNA and then incubated for a further 24 or 48 h under the same conditions, in order to achieve effective gene knockdown.

After the incubation 100 μL of Calbryte-520 dye loading solution was added to each well. Plates were incubated for 60 min to allow the dye to enter the cells and bind to intracellular calcium, producing a fluorescent signal.

Fluorescence intensity was measured using a microplate reader with excitation at 490 nm and emission at 525 nm. Results were expressed as relative fluorescence units (RFU) normalized to control.

### 2.5 Immunofluorescence

MDA-MB321 cells grown on glass slides in 4% paraformaldehyde for 20 min and permeabilized with 0.1% Triton X-100 (Sigma Aldrich, Steinheim, Germany) for 4 min. The slides were then incubated with rabbit polyclonal anti-hLFG antibody (1:100 dilution) and mouse polyclonal anti-h-ItdIns3,4,5P3 antibody (Invitrogen, Carlsbad, CA, USA) at 37°C for 1 h. Slides were then washed three times with ice-cold PBS and incubated with Alexa Fluor 488- and Alexa Fluor 680-conjugated goat anti-rabbit and anti-mouse secondary antibodies (1:600 dilution each; Invitrogen, USA) at 37°C for 30 min. After three additional washes with PBS, the samples were dried and mounted with Vectashield mounting medium (Vector Laboratories, Burlingame, CA) containing the DNA staining dye DAPI (4′,6′-diamidino-2-phenylindole).

Images were captured using a ZEISS Axiovert 200 M fluorescence microscope equipped with the appropriate barrier filters. This image provides an exemplary representation of the intracellular localization of the investigated proteins.

### 2.6 Lipid membranes

The Interaction of LFG with cellular membranes, specifically lipids, was assessed with the use of Membrane Lipid Strips™ (Product P-6002) purchased from Echelon Biosciences Inc (Salt Lake City, UT, USA). The 2 × 6 cm hydrophobic membranes were already prepared with 15 spots of different biologically active lipids, that can typically be found in cell membranes, each spot containing 100 pmol of the specific lipid.

In addition to the Membrane Lipid Strip, the interaction of LFG with lipids in the cellular membrane was also investigated with the use of PIP Array™ (Product P-6100) purchased from Echelon Biosciences Inc. The 4 × 5 cm hydrophobic membranes had been prespotted with eight different lipids with a concentration gradient of seven different concentrations, therefore allowing not only the examination of protein-lipid interaction but also the degree of the binding.

The prespotted Membrane Lipid Strips™ and PIP Array™ were used following the given protocol for PIP Strip™ membrane-type products from Echelon Biosciences with the Buffer being PBS + 3% BSA locking Solution and the added protein of interest being Lifeguard. The final detection of possible binding and interaction between the prespotted lipids and LFG was determined through infrared detection using the Li-Cor System. Signal intensities were subsequently quantified using ImageJ software in order to determine the relative binding strength of LFG to the different lipid species.

## 3 Results

### 3.1 Interaction between the lifeguard protein and lipids

To investigate potential interactions between LFG and membrane lipids, we performed membrane lipid strip and phosphoinositide (PIP) array assays. These assays were used to screen for potential LFG-associated signals with different lipid species under the experimental conditions employed. The Membrane Lipid Strip provided an overview of potential LFG–lipid binding signals (Fig. 1A), whereas the PIP array enabled the assessment of phosphoinositide-associated signal patterns across a range of lipid concentrations (Fig. 1B).

**Fig. 1:**
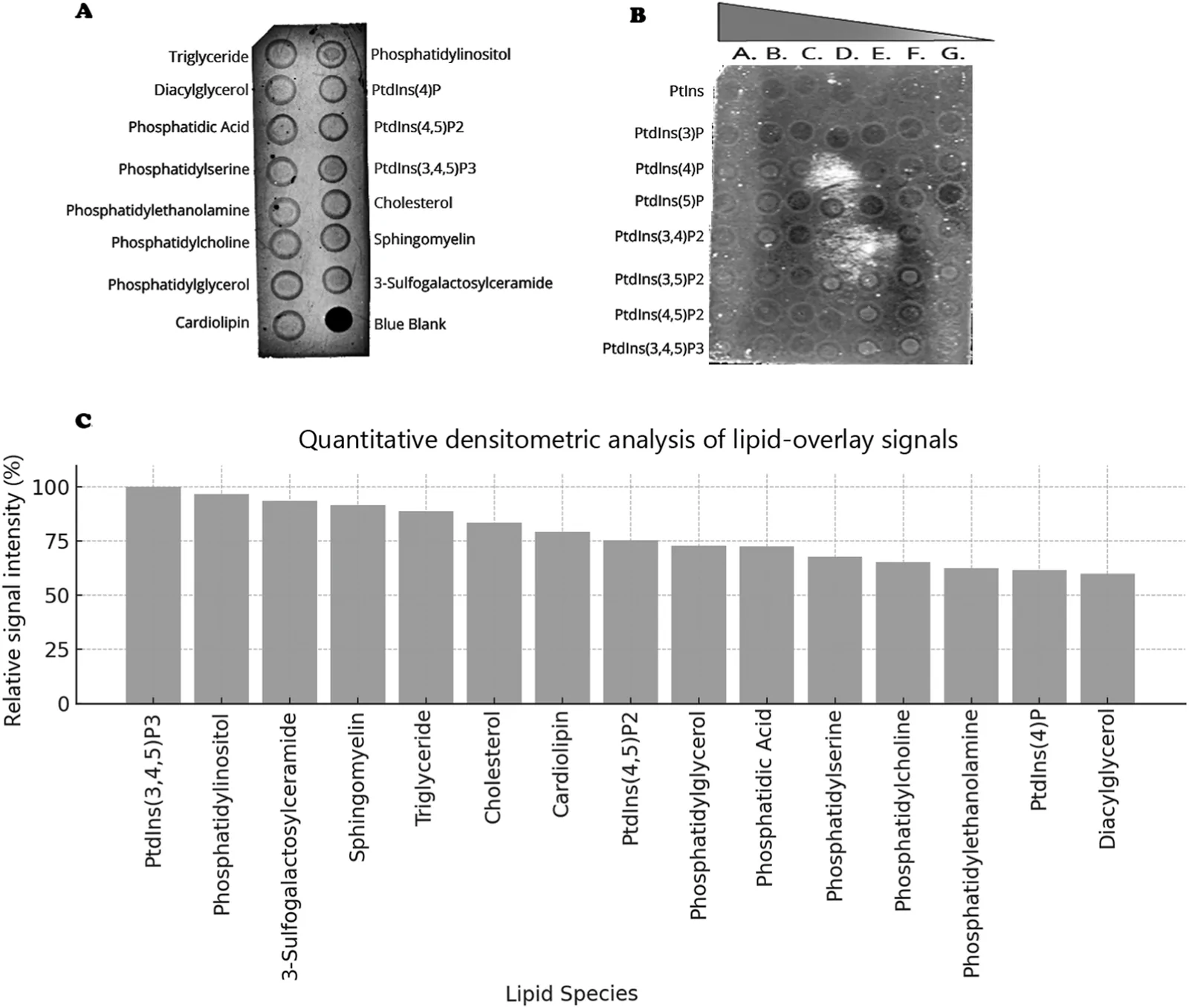
Screening of potential LFG–lipid interactions using membrane lipid strip and phosphoinositide (PIP) array assays. (A) Membrane lipid strip assay showing binding signals of recombinant LFG to different biologically active lipid species immobilized on a nitrocellulose membrane. The assay was used as a qualitative screening method to assess potential lipid-binding preferences under the experimental conditions employed. (B) Phosphoinositide (PIP) array showing binding signals of LFG to eight phosphoinositide species spotted at seven decreasing lipid concentrations (left to right): A, 100 pmol/spot; B, 50 pmol/spot; C, 25 pmol/spot; D, 12.5 pmol/spot; E, 6.25 pmol/spot; F, 3.13 pmol/spot; G, 1.56 pmol/spot. (C) Densitometric analysis of the membrane lipid strip shown in (A). Because signal detection was based on a colorimetric reaction producing dark spots, the image was inverted prior to densitometric analysis so that stronger binding signals corresponded to higher intensity values. The quantification shown represents the analysis of the membrane lipid strip presented in panel (A).

The membrane lipid strip assay revealed detectable signals for several lipid species, including prominent signals for phosphatidylinositol and PtdIns(3,4,5)P_3_ (Fig. 1A). Densitometric analysis of relative signal intensities (Fig. 1C) based on inverted colorimetric signals revealed the highest signal intensities for PtdIns(3,4,5)P_3_ and phosphatidylinositol, followed by 3-sulfogalactosylceramide and sphingomyelin. Moderate signal intensities were observed for neutral lipids and cardiolipin, whereas diacylglycerol and zwitterionic phospholipids displayed weaker signals. Overall, the lipid-overlay assay produced detectable signals associated with several phosphoinositides and other lipid species, with differences in relative signal intensity between individual lipids under the experimental conditions employed (Fig. 1A–C).

The PIP array demonstrated detectable LFG-associated signals for multiple phosphoinositide species, including phosphatidylinositol, PtdIns(4)P, PtdIns(5)P, PtdIns(3,4)P_2_, PtdIns(3,5)P_2_, PtdIns(4,5)P_2_ and PtdIns(3,4,5)P_3_. The signal intensities varied across the lipid concentrations tested. PtdIns(4)P, PtdIns(5)P and PtdIns(3,4)P_2_ produced detectable signals primarily at higher lipid concentrations, whereas PtdIns, PtdIns(3,5)P_2_, PtdIns(4,5)P_2_ and PtdIns(3,4,5)P_3_ produced detectable signals also at lower lipid concentrations. Among the phosphoinositides tested, PtdIns(3,5)P_2_ and PtdIns(3,4,5)P_3_ displayed detectable signals across a broader range of the lipid concentrations tested. As lipid-overlay assays represent qualitative screening methods, these findings should be interpreted as evidence of potential lipid-binding preferences under the experimental conditions employed and do not by themselves establish specific or physiologically relevant LFG–phosphoinositide interactions in cellular membranes.

### 3.2 Spatial distribution of LFG and PtdIns(3,4,5)P_3_ in MDA-MB-231 cells

Cells were labeled with antibodies against LFG and phosphatidylinositol-3,4,5-trisphosphate (PtdIns(3,4,5)P_3_), a phosphoinositide involved in PI3K-dependent intracellular signaling pathways [52]. PtdIns(3,4,5)P_3_ displayed a predominantly perinuclear and intracellular membrane-associated staining pattern, while LFG exhibited a partially overlapping intracellular staining pattern, with prominent signals in perinuclear regions and intracellular membrane-associated areas. Overlay of both channels revealed areas of partial spatial overlap between LFG and PtdIns(3,4,5)P_3_ signals, particularly in perinuclear regions and intracellular membrane-associated areas (Fig. 2D).

**Fig. 2:**
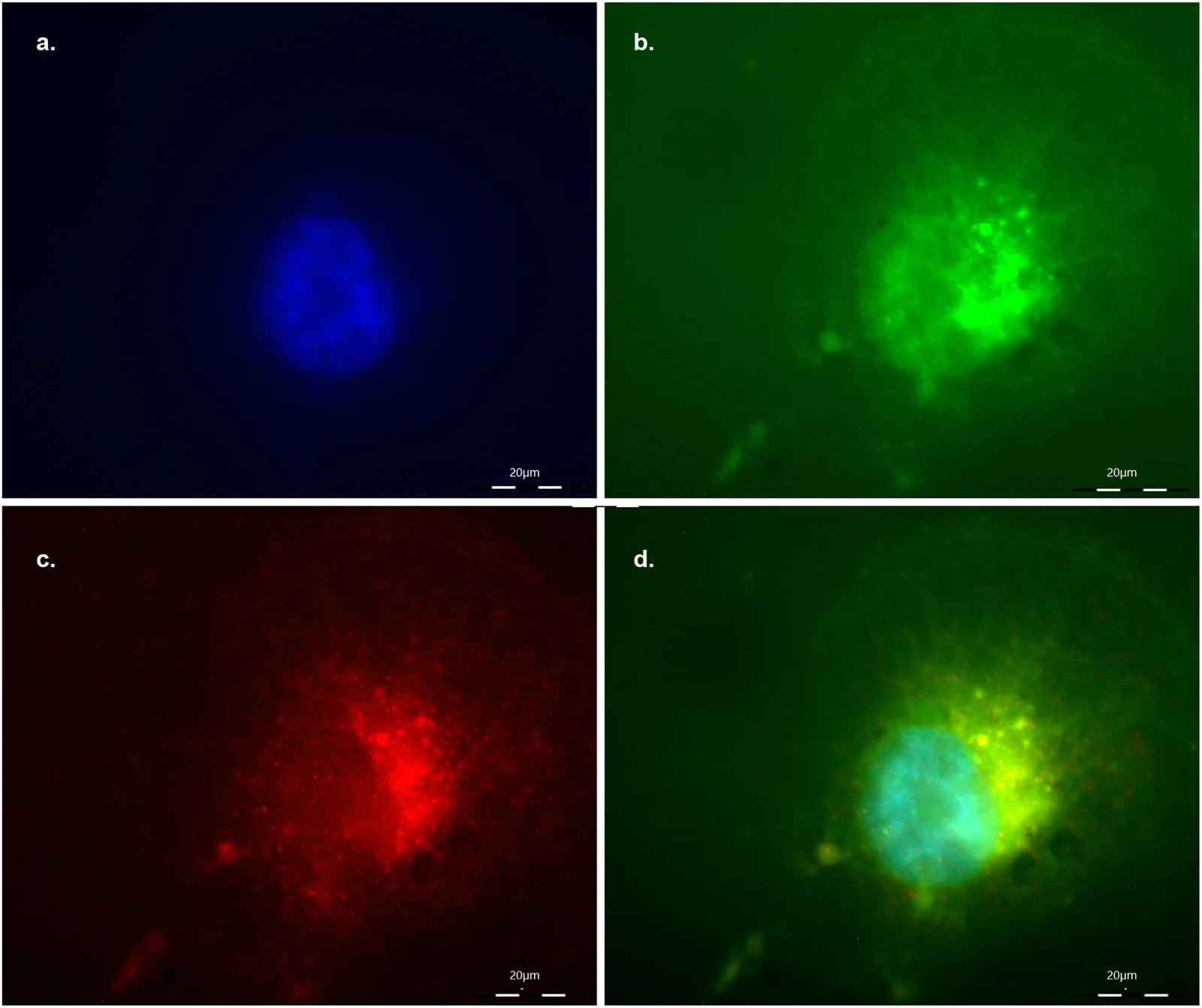
**Immunofluorescence analysis of the intracellular distribution of LFG and phosphatidylinositol-3,4,5-trisphosphate (PtdIns(3,4,5)P_3_) in MDA-MB-231** breast cancer cells. (A) Cell nuclei stained with DAPI (blue). (B) Cells stained with a mouse anti-PtdIns(3,4,5)P_3_ antibody (green), indicating the intracellular distribution of PtdIns(3,4,5)P_3_. (C) Cells stained with a rabbit anti-LFG antibody (red), showing the intracellular localization of LFG. (D) Merged fluorescence image. Areas of partial spatial overlap between LFG and PtdIns(3,4,5)P_3_ fluorescence signals appear yellow/orange. The images represent qualitative observations only; quantitative colocalization analysis and organelle-specific markers were not included in the present study.

Although these qualitative observations do not permit assignment of the observed signal overlap to a specific intracellular organelle or membrane compartment, they demonstrate areas of partial overlap between the LFG and PtdIns(3,4,5)P_3_ fluorescence signals under the imaging conditions employed. Because organelle-specific markers and quantitative colocalization analyses were not included in the present study, these observations should be interpreted as qualitative evidence of partial spatial overlap rather than definitive colocalization. Future studies employing organelle-specific markers together with quantitative colocalization analyses will be required to determine whether LFG and PtdIns(3,4,5)P_3_ occupy shared subcellular compartments and to further characterize their spatial relationship.

### 3.3 Western blot/Ca^2+^ assay

To investigate the effect of siRNA-mediated silencing on LFG (FAIM2) protein expression, MDA-MB-231 cells were transfected with either a non-targeting control siRNA or **an** LFG-specific siRNA and analyzed after 24 and 48 h. Protein levels were determined by Western blotting and densitometric analysis using ImageJ. LFG protein levels were normalized to β-tubulin as a loading control.

Compared with untreated cells and non-targeting control siRNA-transfected cells, LFG protein expression was reduced in a time-dependent manner following transfection with LFG-specific siRNA. After 24 h of LFG siRNA treatment, LFG expression was reduced by approximately 15%, whereas after 48 h, a more pronounced reduction of approximately 40% was observed (Fig. 3A and B). In contrast, transfection with non-targeting control siRNA had no detectable effect on LFG protein expression, supporting successful reduction of LFG protein expression by the LFG-specific siRNA under the experimental conditions employed.

**Fig. 3:**
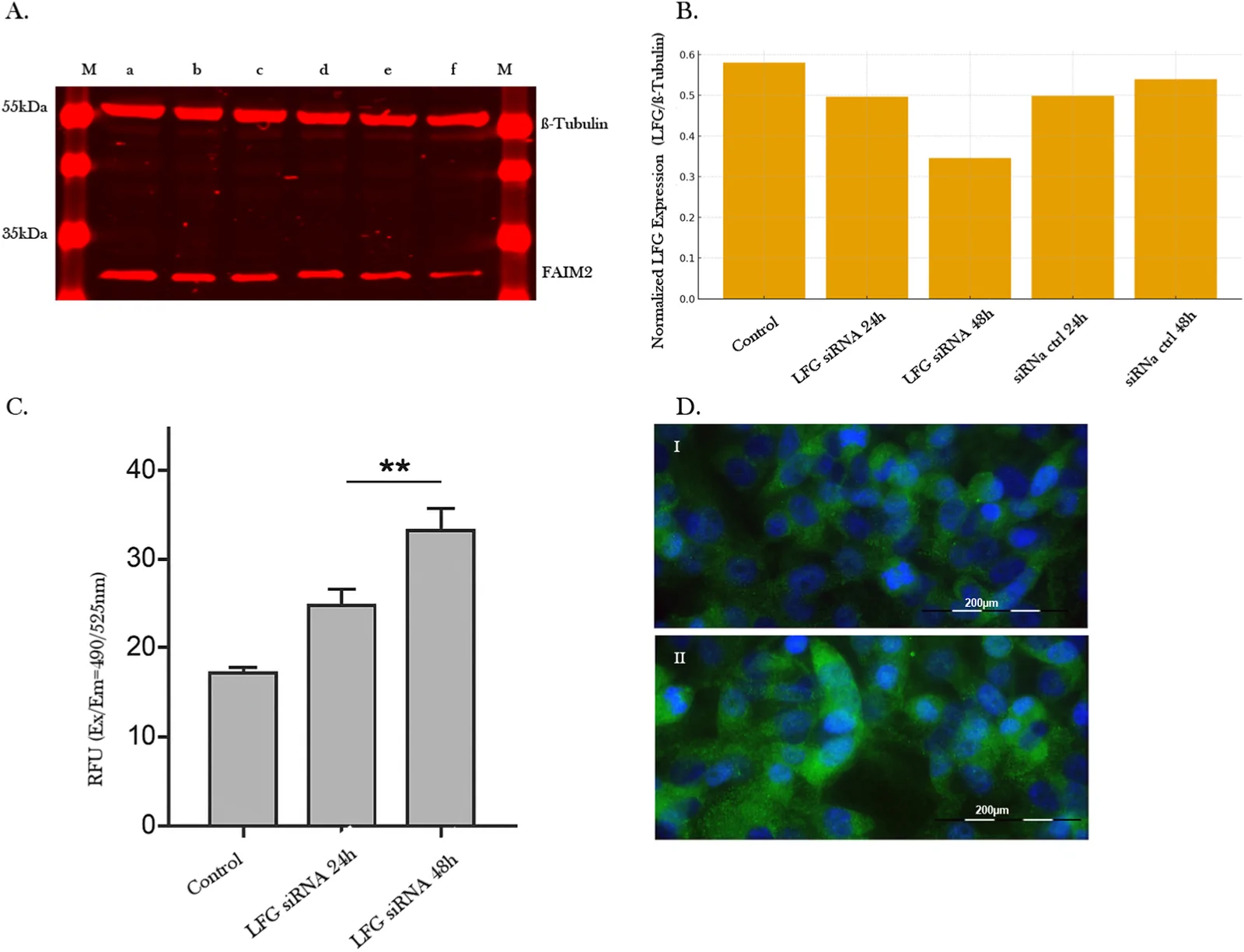
Effects of siRNA-mediated LFG knockdown on LFG protein expression and intracellular calcium-associated fluorescence in MDA-MB-231 breast cancer cells. (A) Western blot analysis of LFG protein expression following transfection with LFG-specific siRNA or non-targeting control siRNA for 24 and 48 h. Total protein lysates were separated by SDS-PAGE, transferred to PVDF membranes, and probed with anti-LFG and anti-β-tubulin antibodies. Lanes: a, untreated control; b, non-targeting control siRNA (24 h); c, non-targeting control siRNA (48 h); d, untreated control; e, LFG-specific siRNA (24 h); f, LFG-specific siRNA (48 h). (B) Densitometric quantification of LFG protein expression normalized to β-tubulin. LFG protein expression was reduced by approximately 40% after 48 h of LFG-specific siRNA treatment (p < 0.001). (C) Measurement of intracellular calcium-associated fluorescence using the fluorescent calcium indicator Calbryte-520 (RFU; Ex/Em = 490/525 nm) following LFG knockdown. Calcium-associated fluorescence increased 2.3 ± 0.4-fold after 48 h of LFG-specific siRNA treatment (p < 0.01). Because Calbryte-520 does not distinguish between different intracellular calcium pools or calcium sources, the measurements represent total intracellular calcium-associated fluorescence rather than calcium originating from a specific intracellular compartment. (D) Representative fluorescence microscopy images of intracellular calcium-associated fluorescence. I, untreated control cells; II, cells transfected with LFG-specific siRNA for 48 h.

To investigate whether LFG downregulation was associated with alterations in intracellular calcium homeostasis, intracellular calcium-associated fluorescence was measured 24 and 48 h post-transfection using a fluorescence-based assay. Relative fluorescence units (RFU; Ex/Em: 490/525 nm) increased in a time-dependent manner following LFG downregulation (Fig. 3C). After 48 h of LFG siRNA treatment, calcium-associated fluorescence increased 2.3 ± 0.4-fold compared with control cells (p < 0.01). Representative fluorescence microscopy images (Fig. 3D) showed a corresponding increase in fluorescence intensity. Image I represents untreated control cells, while image II shows LFG siRNA-transfected cells at 48 h. Cells transfected with LFG siRNA exhibited increased intracellular green fluorescence intensity, in agreement with the plate-reader measurements.

Because the fluorescence-based assay does not distinguish between different sources or cellular compartments of calcium, the observed signals should be interpreted as overall intracellular calcium-associated fluorescence. Therefore, these measurements demonstrate an association between LFG downregulation and altered intracellular calcium-associated fluorescence but do not establish whether the observed change reflects ER calcium release, extracellular calcium influx, mitochondrial calcium handling, or other alterations in intracellular calcium homeostasis.

## 4 Discussion

Breast cancer remains the most commonly diagnosed malignancy in women worldwide, requiring ongoing research to elucidate its underlying molecular mechanisms and identify novel therapeutic targets [1,2]. A critical aspect of oncology research is to investigate the dysregulation of intracellular signaling pathways that control apoptosis and tumor cell survival [6,11,52,53]. One such regulatory component is Lifeguard (LFG), an anti-apoptotic transmembrane protein that has been implicated in enhancing tumor cell resistance to programmed cell death. While the structural characteristics and subcellular localization of LFG have been well characterized, the precise molecular mechanisms by which LFG contributes to apoptotic inhibition, particularly in the context of its overexpression in cancer cells, remain to be fully elucidated [13,16,17,18,19,21].

Given the membrane-associated localization of LFG, we hypothesized that LFG may interact with phosphoinositides, which are important regulators of intracellular signaling, membrane organization and calcium homeostasis [[39], [40], [41], [42], [43], [44], [45], [46]]. In addition to their well-established roles at the plasma membrane, phosphoinositides contribute to membrane identity, organelle communication and intracellular signaling across multiple cellular compartments [43,54]. Using membrane lipid strips and PIP arrays, we observed differential LFG-associated signals for several phosphoinositides, with prominent signals for phosphatidylinositol and PtdIns(3,4,5)P_3_. These findings indicate potential lipid-binding preferences under the experimental conditions employed rather than establishing specific physiological LFG–phosphoinositide interactions.

However, lipid-overlay assays are widely regarded as qualitative screening methods because lipids are presented on nitrocellulose membranes under highly artificial conditions that do not fully reproduce the organization, lateral mobility, and biophysical properties of biological membranes [55,56]. Consequently, the observed signals should be interpreted as exploratory and preliminary evidence of lipid-binding preferences rather than definitive proof of physiological membrane binding. Additional orthogonal approaches, including liposome-binding assays, co-immunoprecipitation, surface plasmon resonance or other quantitative biophysical methods, will be required to validate the potential interactions under more physiological conditions. Accordingly, lipid-overlay assays are generally considered hypothesis-generating approaches that require confirmation by complementary quantitative methods [55,56]. Furthermore, the relatively broad signal pattern observed for several negatively charged lipid species may reflect either biologically relevant lipid recognition or, at least in part, electrostatic interactions, which represent a recognized limitation of lipid-overlay assays [55,56].

Interestingly, the observed lipid-associated signal pattern is broadly consistent with previous reports demonstrating that several membrane-associated proteins preferentially associate with phosphoinositide-rich membrane domains despite lacking classical phosphoinositide-binding motifs such as pleckstrin homology (PH) domains [57,58]. In addition, detectable LFG-associated signals were also observed for sulfated glycosphingolipids and sphingomyelin. Similar binding patterns have been described for proteins associated with lipid raft microdomains [59]. Previous studies have shown that LFG localizes to lipid raft-associated membrane domains in neuronal cells [24]. However, this hypothesis requires confirmation by additional localization studies employing higher-resolution imaging and organelle-specific markers.

To further investigate the potential biological relevance of the lipid-overlay findings, we examined the intracellular distribution of LFG and PtdIns(3,4,5)P_3_ by immunofluorescence microscopy. While phosphoinositides are predominantly localized to the plasma membrane, distinct phosphoinositide species are also present in several intracellular membrane compartments, including endosomes, the Golgi apparatus, and other intracellular membranes, where they contribute to membrane trafficking and intracellular signaling [[60], [61], [62]].

The observed partial spatial overlap between LFG and PtdIns(3,4,5)P_3_ is consistent with a possible spatial association of the two signals within intracellular membrane-associated regions but does not establish a functional interaction between LFG and PtdIns(3,4,5)P_3_. Because organelle-specific markers were not included in the present study, the observed overlap cannot be assigned to a specific intracellular structure, such as the endoplasmic reticulum (ER) or ER membrane contact sites (MCS). Previous studies have demonstrated that phosphoinositides are present in several intracellular membrane compartments, including membrane contact sites, where they contribute to the regulation of lipid metabolism, intracellular calcium signaling and membrane dynamics through the coordinated activity of phosphoinositide kinases and phosphatases [22,[60], [61], [62], [63], [64], [65], [66], [67], [68]]. Whether LFG participates in these processes remains to be determined and requires further investigation using higher-resolution imaging and organelle-specific markers. Taken together, the lipid-overlay findings and the observed partial spatial overlap raise the possibility that LFG may be associated with phosphoinositide-related signaling processes. However, these observations do not demonstrate that LFG regulates phosphoinositide-dependent calcium signaling. The present study does not directly demonstrate modulation of phosphoinositide metabolism, PLC activity, IP3 production or IP3 receptor activation. Therefore, these mechanisms should be regarded as hypotheses requiring future experimental validation.

Under physiological conditions, phosphatidylinositol 4,5-bisphosphate (PtdIns(4,5)P_2_) serves as the principal substrate for phospholipase C (PLC), generating inositol 1,4,5-trisphosphate (IP_3_) and diacylglycerol (DAG), whereas phosphatidylinositol 3,4,5-trisphosphate (PtdIns(3,4,5)P_3_) participates in distinct but interconnected phosphoinositide signaling pathways regulating multiple cellular processes [32,47,48,52,54,[60], [61], [62], [63], [64], [65], [66], [67],^69^,^70^].

IP_3_ subsequently binds to its receptor on the endoplasmic reticulum, promoting intracellular Ca^2+^ release. Although our lipid-overlay and immunofluorescence data suggest a potential association between LFG and phosphoinositide-associated signaling, the present study does not demonstrate that LFG directly regulates phosphoinositide metabolism or IP_3_-dependent calcium signaling. Whether LFG directly affects phosphoinositide metabolism, IP_3_ generation, or IP_3_ receptor-mediated calcium signaling remains to be investigated in future mechanistic studies.

The present study showed that siRNA-mediated downregulation of LFG in MDA-MB-231 breast cancer cells resulted in a time-dependent reduction in LFG protein expression, with a moderate decrease after 24 h and a more pronounced reduction of approximately 40% after 48 h. Although complete depletion of LFG was not achieved, the approximately 40% reduction in LFG protein expression was associated with increased intracellular calcium-associated fluorescence under the experimental conditions employed. Because no rescue experiment with an siRNA-resistant LFG construct was performed, the present data cannot fully exclude secondary or off-target effects of the siRNA treatment as contributors to the observed calcium-associated phenotype. The incomplete knockdown efficiency observed here is consistent with previous reports describing the relatively high transfection resistance and variable siRNA efficiency in MDA-MB-231 cells [71]. The observed time-dependent reduction in LFG protein levels confirms successful siRNA-mediated knockdown under the experimental conditions employed in this study.

Our fluorescence-based calcium assay showed an increase in intracellular calcium-associated fluorescence following LFG knockdown. However, because the assay does not distinguish between ER-derived calcium release, cytosolic calcium accumulation, mitochondrial calcium uptake, or extracellular calcium influx, our data should be interpreted as evidence of altered intracellular calcium homeostasis rather than direct proof of ER calcium release. More specifically, the present assay does not allow identification of the cellular source or transport mechanism responsible for the observed increase in calcium-associated fluorescence.

These observations can be considered in the context of previous studies describing calcium-regulatory functions of other members of the transmembrane Bax inhibitor motif-containing (TMBIM) family, particularly Bax inhibitor-1 (BI-1), which has been reported to influence ER calcium leakage and thereby modulate apoptosis susceptibility [68,72]. More broadly, members of the TMBIM protein family have emerged as important regulators of intracellular calcium homeostasis and cellular stress responses, although the molecular mechanisms differ between individual family members [38]. Therefore, comparisons between LFG and BI-1 should be interpreted cautiously, as calcium-regulatory mechanisms described for one TMBIM family member cannot necessarily be assumed to apply to another. BI-1 overexpression has been associated with decreased cytosolic Ca^2+^ concentrations due to altered ER Ca^2+^ permeability, thereby reducing IP_3_ receptor-mediated calcium release during apoptotic signaling [73,74].

A direct interaction between LFG and IP_3_R was not investigated in the present study. Accordingly, the current data do not allow conclusions regarding direct regulation of IP_3_R-mediated calcium signaling by LFG. Nevertheless, the lipid-overlay findings and the observed partial spatial overlap with PtdIns(3,4,5)P_3_ raise the possibility that LFG may participate in phosphoinositide-associated signaling pathways. However, the present study does not establish the underlying molecular mechanisms, and any mechanistic relationship therefore remains speculative and requires future experimental validation.

In summary, this exploratory study provides initial evidence of LFG-associated signals with several phosphoinositides under lipid-overlay assay conditions and demonstrates that reduced LFG expression is associated with increased intracellular calcium-associated fluorescence in MDA-MB-231 breast cancer cells. Together, these findings support further investigation of a potential relationship between LFG, phosphoinositide-associated signaling and intracellular calcium homeostasis. However, the present data do not establish a mechanistic or causal relationship linking phosphoinositide association, intracellular calcium regulation, and apoptosis. Additional studies employing complementary lipid-binding approaches, quantitative colocalization analyses, organelle-specific calcium measurements, rescue experiments, and apoptosis assays will be necessary to clarify the functional role of LFG in these signaling pathways.

The present findings should be interpreted in light of the qualitative nature of the lipid-overlay and imaging assays, the non-compartment-specific calcium readout, the incomplete knockdown without a rescue experiment, and the use of a single breast cancer cell line. Despite these limitations, our findings provide preliminary insight into the potential relationship between LFG, phosphoinositides, and intracellular calcium homeostasis and provide a basis for future studies investigating the role of LFG in breast cancer biology. Furthermore, validation in additional breast cancer cell lines and non-tumorigenic mammary epithelial cells will be important to determine the generalizability of the present findings.

## CRediT authorship contribution statement

**Anna Marie Welzel:** Writing – review & editing, Writing – original draft, Visualization, Methodology, Investigation, Formal analysis, Data curation, Conceptualization. **Lena Maria Herrmann:** Investigation. **Nadjib Dastagir:** Writing – review & editing. **Sarah Strauß:** Writing – review & editing, Resources. **Peter M. Vogt:** Writing – review & editing, Supervision, Funding acquisition. **Vesna Bucan:** Writing – review & editing, Visualization, Validation, Supervision, Resources, Project administration, Methodology, Investigation, Funding acquisition, Formal analysis, Data curation, Conceptualization.

## Consent for publication

All authors have given their permission.

## Availability of data and materials

All provided data and materials are available.

## Funding

This research received no external funding.

## Declaration of competing interest

The authors declare that they have no known competing financial interests or personal relationships that could have appeared to influence the work reported in this paper.

## Figures and Tables

**figs1:**
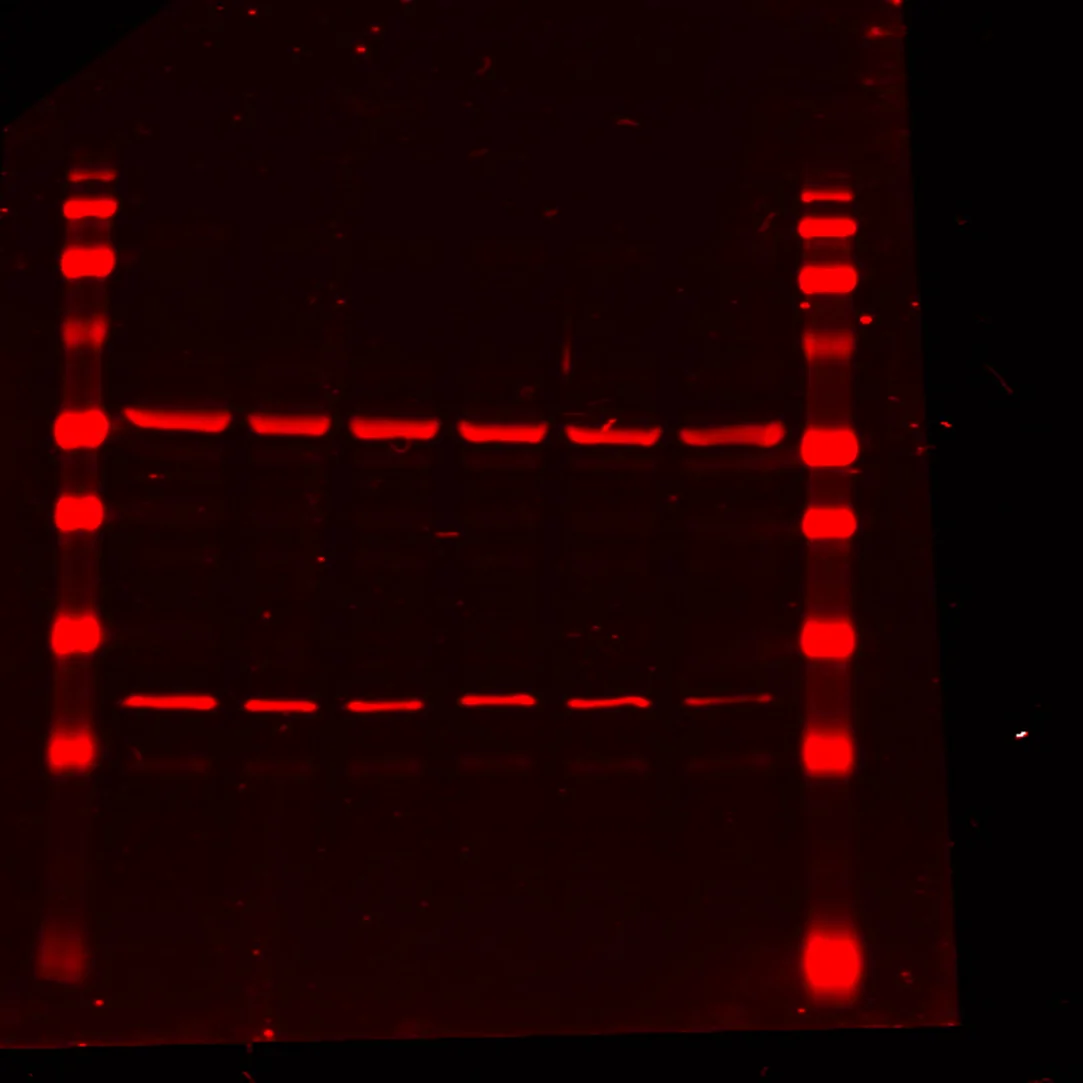


## Data Availability

Data will be made available on request.
